# A Case of Graves’ Disease Exacerbation Triggered by BNT162b2 Vaccination

**DOI:** 10.7759/cureus.84863

**Published:** 2025-05-26

**Authors:** Hideyuki Iwamoto, Tomohiko Kimura, Erina Nakao, Fuminori Tatsumi, Hideaki Kaneto

**Affiliations:** 1 Department of Diabetes, Endocrinology and Metabolism, Kawasaki Medical School, Kurashiki, JPN

**Keywords:** bnt162b2 vaccine, covid-19, covid-19 vaccination, graves´disease, thiamazole

## Abstract

Graves' disease is an autoimmune thyroid disorder characterized by the production of thyroid-stimulating hormone receptor antibodies and is one of the leading causes of hyperthyroidism. While both genetic and environmental factors are involved in its onset, the detailed mechanisms remain unclear. The COVID-19 pandemic led to the development and global distribution of mRNA vaccines, such as BNT162b2, to combat SARS-CoV-2. However, concerns have arisen regarding the potential exacerbation of autoimmune diseases following vaccination. This report presents a case of a 48-year-old male with Graves' disease, treated with 2.5 mg/day of thiamazole (MMI), who developed symptoms such as fatigue, hand tremors, and exertional dyspnea on the seventh day after receiving the first dose of the BNT162b2 vaccine. Laboratory findings confirmed severe thyrotoxicosis, and he was diagnosed with an exacerbation of Graves' disease. His condition improved with an increased MMI dosage and supportive therapy. The possible mechanisms for Graves' disease exacerbation include molecular mimicry between the SARS-CoV-2 spike protein and thyroid antigens, as well as immune activation by vaccine adjuvants. Several recent reports, including this case, highlight the importance of careful monitoring of patients with pre-existing autoimmune diseases after vaccination. This case underscores the need for early diagnosis and prompt intervention in managing post-vaccination autoimmune disease exacerbations. Further research is required to clarify the causal relationship between vaccines and autoimmune disease flares, identify risk factors, and establish preventive measures. Additionally, both healthcare providers and patients must remain vigilant for potential health changes following vaccination and seek medical attention promptly.

## Introduction

Graves' disease is an autoimmune thyroid disorder characterized by hyperthyroidism caused by autoantibodies against the thyroid-stimulating hormone (TSH) receptor. It is one of the leading causes of hyperthyroidism. This disease primarily affects the thyroid glands but is also associated with systemic symptoms. It can lead to hyperthyroidism, diffuse goiter, and ophthalmopathy. While the exact cause remains unclear, its onset is believed to involve both genetic and environmental factors, including external factors such as trauma and stress, as well as internal factors such as pregnancy and childbirth [1]. In late 2019, COVID-19 was first reported in Wuhan, China, and quickly spread worldwide. In March 2020, the WHO declared it a pandemic, significantly impacting healthcare systems, economies, and social structures [2,3]. In response, mRNA vaccines were developed to combat SARS-CoV-2, leading to their widespread global use [4]. The mRNA-based COVID-19 vaccines, particularly BNT162b2 (Pfizer-BioNTech), played a crucial role in controlling the pandemic. These vaccines use mRNA encoding the SARS-CoV-2 spike protein, allowing for rapid development and high efficacy [5]. However, their widespread use has raised concerns regarding potential effects on the immune system, particularly their association with the onset and exacerbation of autoimmune diseases [6]. Reports have documented cases of hyperthyroidism occurring within several weeks after mRNA vaccination [7,8], leading to concerns about immune modulation induced by the vaccine. Here, we report a case of Graves' disease exacerbated following BNT162b2 vaccination.

## Case presentation

**Figure 1:**
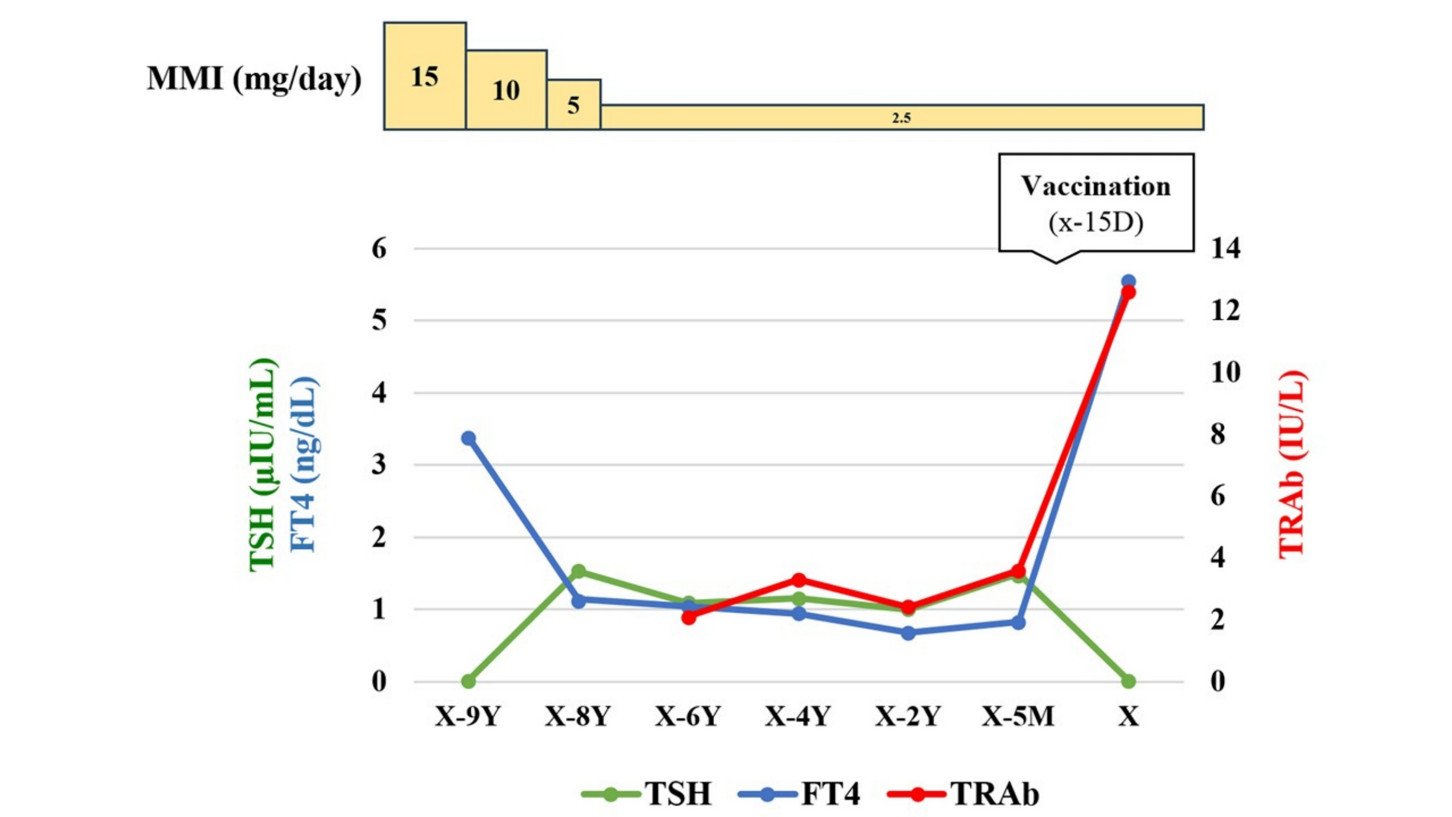
Time course of thyroid function and thiamazole (MMI) dosage before hospitalization. TSH: Thyroid-stimulating hormone; FT4: Free thyroxine; TRAb: Thyroid-stimulating hormone receptor antibody; X: Admission date; Y: Year; M: Month; D: Day.

The patient was a 48-year-old male who presented with exertional dyspnea, hand tremors, and bilateral lower limb edema as his chief complaints. He had been diagnosed with Graves' disease nine years earlier and was initially treated with thiamazole (MMI) at 15 mg/day. After achieving stable thyroid function, his dosage was reduced to 2.5 mg/day eight years ago, and he had been maintaining this dosage since then. However, TSH receptor antibodies (TRAb) remained positive. Fifteen days before admission, the patient received his first dose of the SARS-CoV-2 vaccine (BNT162b2). He remained asymptomatic for the first week post-vaccination, but on the seventh day, he developed fatigue, hand tremors, and exertional palpitations. On the fourteenth day, his palpitations worsened, and he noticed bilateral lower limb edema. The next day, he visited the ED, where thyrotoxicosis was suspected, and he was referred to our department. Figure 1 shows the time course of thyroid function and MMI dosage before admission.

On admission, he was alert and conscious. His blood pressure was 143/78 mmHg, heart rate was 115 bpm, respiratory rate was 20 breaths/min, oxygen saturation was 99%, and body temperature was 37.1°C. On physical examination, the thyroid gland was diffusely enlarged with an elastic consistency, and no tenderness was noted on palpation. Laboratory tests revealed severe hyperthyroidism, with TSH <0.01 µIU/mL, FT3 21.60 pg/mL, and FT4 5.56 ng/mL. Additionally, TRAb was significantly elevated to 12.6 IU/L (Table 1).

**Table 1:** Laboratory data on admission. MCV: Mean corpuscular volume; AST: Aspartate aminotransferase; ALT: Alanine aminotransferase; γ-GTP: γ-Glutamyl transpeptidase; ALP: Alkaline phosphatase; LDH: Lactate dehydrogenase; BUN: Blood urea nitrogen; UA: Uric acid; CRP: C-reactive protein; IP: Inorganic phosphorus; TSH: Thyroid-stimulating hormone; FT3: Free triiodothyronine; FT4: Free thyroxine; TRAb: Thyroid-stimulating hormone receptor antibody.

Test	Value	Reference Range
WBCs	5,730 /μL	4,000-10,000 /μL
Neutrophils	47.50%	40-70%
Lymphocytes	36.80%	20-45%
Monocytes	15.00%	2-8%
Eosinophils	0.50%	1-6%
Basophils	0.20%	0-2%
RBCs	415 × 10⁴ /μL	420-580 × 10⁴ /μL
Hemoglobin	12.3 g/dL	13.0-17.0 g/dL
Hematocrit	37.40%	38-50%
MCV	90.1 fL	80-100 fL
Platelets	22.4 × 10⁴ /μL	15.0-40.0 × 10⁴ /μL
Urinalysis		
pH	6	4.6-8.0
U-protein	(–)	(–)
U-glucose	(–)	(–)
U-occult blood	(–)	(–)
Blood Biochemistry		
Total protein	6.0 g/dL	6.5-8.0 g/dL
Albumin	3.3 g/dL	3.8-5.3 g/dL
Total bilirubin	1.3 mg/dL	0.2-1.2 mg/dL
AST	25 U/L	10-40 U/L
ALT	23 U/L	5-45 U/L
γ-GTP	22 U/L	9-35 U/L
ALP	65 U/L	40-130 U/L
LDH	145 U/L	120-240 U/L
Creatinine	0.72 mg/dL	0.6-1.1 mg/dL
BUN	21 mg/dL	8-22 mg/dL
UA	6.5 mg/dL	3.6-7.0 mg/dL
Glucose	92 mg/dL	70-110 mg/dL
CRP	0.08 mg/dL	< 0.3 mg/dL
Sodium	140 mmol/L	135-145 mmol/L
Potassium	3.9 mmol/L	3.5-5.0 mmol/L
Chloride	106 mmol/L	98-107 mmol/L
IP (Inorganic Phosphorus)	3.9 mg/dL	2.5-4.5 mg/dL
Calcium	8.9 mg/dL	8.5-10.2 mg/dL
Magnesium	1.7 mg/dL	1.6-2.4 mg/dL
Endocrine Examination		
TSH	<0.01 μIU/mL	0.4-4.0 μIU/mL
FT3	21.60 pg/mL	2.3-4.0 pg/mL
FT4	5.56 ng/dL	0.8-1.8 ng/dL
TRAb	12.6 IU/L	<2.0 IU/L

**Figure 2:**
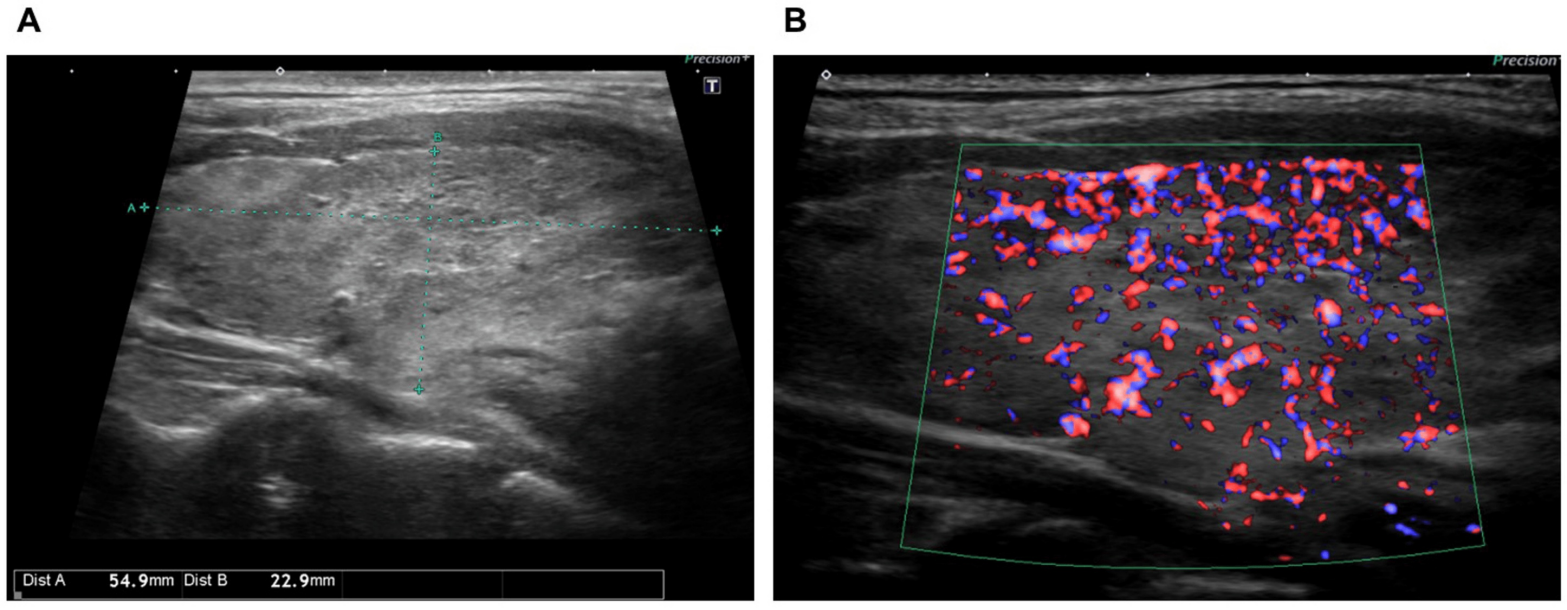
Thyroid ultrasound findings. (A) Thyroid ultrasound showing diffuse thyroid enlargement. (B) Increased blood flow signals within the thyroid.

Thyroid ultrasound showed diffuse thyroid enlargement and increased blood flow signals (Figures 2A-2B), leading to a diagnosis of Graves' disease exacerbation.

**Figure 3:**
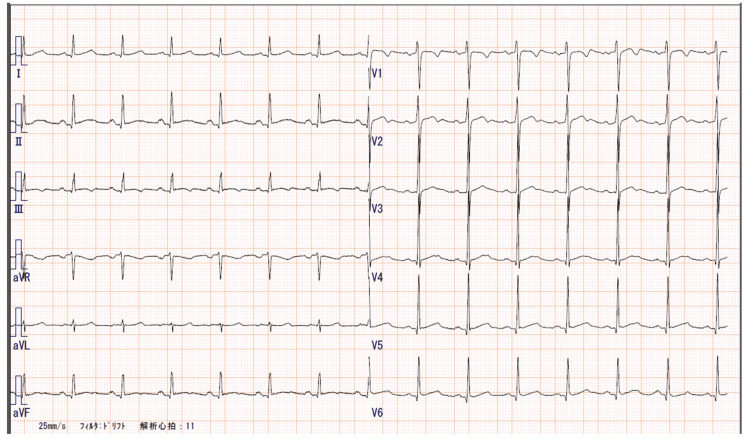
12-lead electrocardiogram. The electrocardiogram shows a normal sinus rhythm with a heart rate of 87 bpm.

**Figure 4:**
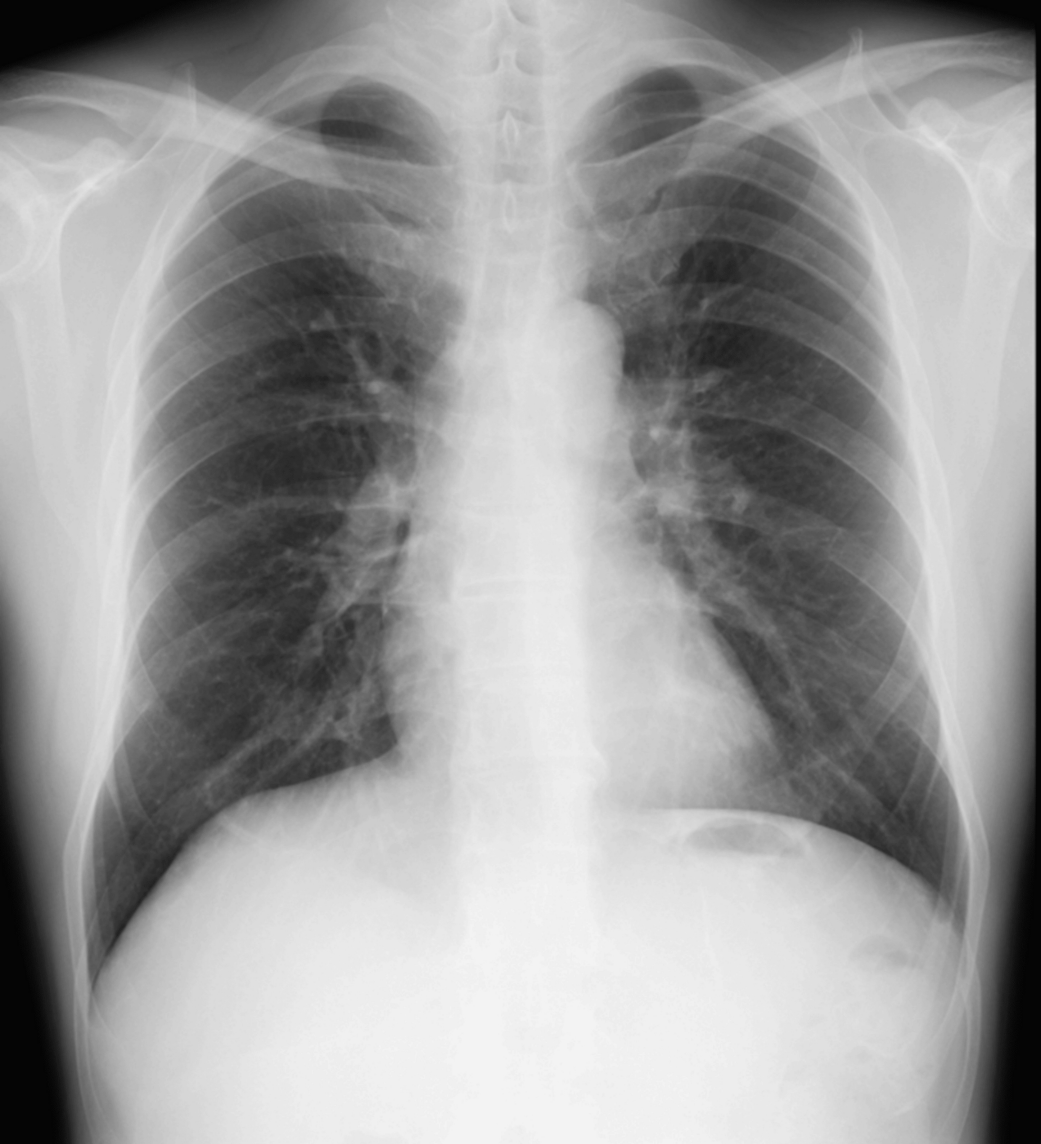
Chest X-ray. The chest X-ray revealed mild cardiomegaly.

A 12-lead electrocardiogram showed a normal sinus rhythm with a heart rate of 87 bpm (Figure 3), but a chest X-ray revealed mild cardiomegaly (Figure 4), and BNP was mildly elevated to 86.5 pg/mL, suggesting potential heart failure.

Given the severe hyperthyroidism and clinical and laboratory findings suggestive of heart failure, we decided to initiate inpatient treatment. The patient was admitted and treated with bed rest, and his MMI dose was increased to 15 mg/day. By the third hospital day, his thyroid function had improved (FT3: 18.61 pg/mL, FT4: 5.30 ng/dL), and his lower limb edema and dyspnea were reduced. Consequently, he was discharged on the fourth hospital day. After discharge, he continued to receive outpatient follow-up at our department for ongoing treatment.

## Discussion

Recent case reports have shown the potential exacerbation of autoimmune diseases following COVID-19 vaccination [9]. Several reports highlight the importance of careful monitoring of patients with pre-existing autoimmune diseases after vaccination. In this case, the patient's symptoms first appeared seven days after receiving the BNT162b2 vaccine, while his thyroid function had been stable prior to vaccination. Additionally, there were no other apparent causes of Graves' disease exacerbation, such as missed medication, smoking, or infection, suggesting a potential link between the vaccination and disease flare-up. Several mechanisms have been proposed for the exacerbation of Graves' disease. One of the most frequently cited theories is adjuvant-induced autoimmune/inflammatory syndrome (ASIA) [10]. Adjuvants are substances used to enhance the immune response to active vaccine components, and some SARS-CoV-2 vaccines contain aluminum or polysorbate 80 as adjuvants. Although BNT162b2 does not contain known adjuvants, cases of Graves' disease triggered by BNT162b2 have met the ASIA diagnostic criteria, suggesting that lipid nanoparticles or polyethylene glycol on their surface may act as adjuvants [11]. Additionally, molecular mimicry has been proposed as a potential contributing factor. For instance, the SARS-CoV-2 spike protein, nucleocapsid protein, and membrane protein have all been found to cross-react with thyroid peroxidase (TPO), which may contribute to the development of autoimmune thyroid diseases [12]. Some case reports have documented severe cases of thyroid storm occurring shortly after BNT162b2 vaccination, emphasizing the importance of close monitoring, particularly in patients with pre-existing autoimmune diseases [13]. On the other hand, similar mechanisms have been reported in other autoimmune diseases induced or exacerbated by COVID-19 vaccination, such as autoimmune hepatitis, Guillain-Barré syndrome, IgA nephropathy, and systemic lupus erythematosus [14,15]. These findings suggest that the observed immune activation may not be specific to Graves' disease but may reflect common immunological pathways shared by autoimmune diseases in general. Future challenges include conducting prospective studies to clarify the relationship between COVID-19 vaccination and autoimmune disease exacerbation, investigating genetic factors and the influence of pre-existing autoimmune conditions, and developing screening protocols for high-risk populations before vaccination.

## Conclusions

This case report describes an exacerbation of Graves' disease triggered by BNT162b2 vaccination. We believe it underscores the importance of early diagnosis and prompt intervention in managing exacerbations of autoimmune diseases following vaccination. Additionally, it is essential for both healthcare providers and patients with autoimmune conditions to recognize that mRNA vaccines may exacerbate underlying diseases. Patients should be advised to monitor their health closely after vaccination and seek medical attention promptly if any symptoms arise.
